# Cognitive profile of patients with and without speech impairment in Parkinson's disease

**DOI:** 10.1590/1980-5764-DN-2022-0093

**Published:** 2023-11-20

**Authors:** Nariana Mattos Figueiredo Sousa, Juliana de Fátima Garcia Diniz, Ana Paula Galvão, Sonia Maria Dozzi Brucki

**Affiliations:** 1 Rede SARAH de Hospitais de Reabilitação, Salvador BA, Brazil.; 2 Universidade de São Paulo, Faculdade de Medicina, Departamento de Neurologia, São Paulo SP, Brazil.

**Keywords:** Speech Disorders, Neurocognitive Disorders, Neuropsychological Tests, Auditory Perception, Parkinson Disease, Distúrbios da Fala, Transtornos Neurocognitivos, Testes Neuropsicológicos, Percepção Auditiva, Doença de Parkinson

## Abstract

Cognitive functions have been the subject of studies evaluating the pathophysiological mechanism of speech control.

**Objective:**

To compare the groups of patients with and without speech disorders with cognitive assessment, demographic, and clinical data (disease duration, functionality, and motor symptoms).

**Methods:**

Retrospective, cross-sectional study. Patients were evaluated using the Addenbrooke's Cognitive Examination III and neuropsychological tests. The following speech subsystems were analyzed: articulation, phonation, resonance, and prosody, through auditory-perceptual evaluation (based on the Protocol for the Evaluation of Acquired Speech Disorders in Individuals with Parkinson's Disease — PADAF Protocol tests), observing aspects of speech programming and execution. The patients were distributed into three subgroups (normal cognition, mild cognitive impairment, and dementia). After speech evaluation, they were divided into two subgroups (with and without speech disorders).

**Results:**

A total of 150 patients participated in this study, 104 men and 46 women, 63.58 (8.81) years of age, 11.03 (4.00) years of schooling, 6.61 (4.69) years of disease progression, and with the highest proportion of individuals in stage I–II of the Hoehn & Yarh (H&Y) scale (86, or 57.33%). Statistically significant differences were observed between subgroups with and without speech alteration. Worse performance was verified in the Trail Making Test (TMT) TMT-Δ and a tendency of difference in the TMT-B of the subgroup with speech disorders, in addition to worse severity of motor symptoms (H&Y) and cognitive complaints.

**Conclusion:**

Individuals with speech disorders brought more frequent cognitive complaints and impairment below expected in tests assessing executive functions. Future studies, with stratification by type of speech disorder, are necessary to contribute to and validate these results.

## RESUMO.

As funções cognitivas têm sido alvo de estudos que avaliam o mecanismo patofisiológico do controle da fala.

**Objetivo:**

Comparar subgrupos de pacientes com e sem alterações de fala quanto à avaliação cognitiva, dados demográficos e clínicos (tempo de evolução da doença, funcionalidade e gravidade dos sintomas motores).

**Métodos:**

Estudo retrospectivo, de corte transversal. Os pacientes foram avaliados pelo Exame Cognitivo de Addenbrooke III e testes neuropsicológicos. Foram analisados os seguintes subsistemas da fala: articulação, fonação, ressonância e prosódia, por meio de avaliação perceptivo-auditiva (baseada em testes do Protocolo de Avaliação dos Distúrbios Adquiridos de Fala em Indivíduos com Doença de Parkinson — PADAF), sendo observados aspectos da programação e execução da fala. Os pacientes foram distribuídos em três subgrupos (cognição normal, comprometimento cognitivo leve e demência). Após a avaliação da fala, foram divididos em dois subgrupos (com desordens da fala e sem desordens da fala).

**Resultados:**

Participaram deste estudo 150 pacientes, 104 homens e 46 mulheres, com 63,58 (8,81) anos de idade, 11,03 (4,00) anos de escolaridade e 6,61 (4,69) anos de evolução da doença, e maior proporção de indivíduos no estágio I–II da Escala de Hoehn & Yarh — H&Y (86, ou 57,33%). Foram observadas diferenças estatisticamente significantes entre os subgrupos com e sem alteração da fala. Houve pior desempenho no Trail Making Test (TMT) TMT-Δ e tendência de diferença no TMT-B no subgrupo com desordens da fala, além de pior gravidade dos sintomas motores (H&Y) e queixa cognitiva.

**Conclusão:**

Os indivíduos com desordens da fala trouxeram queixas cognitivas com maior frequência e prejuízo abaixo do esperado nos testes que avaliam as funções executivas. Estudos futuros, com estratificação por tipo de distúrbio da fala, são necessários para a contribuição e validação destes resultados.

## INTRODUCTION

Parkinson's disease (PD) is a neurodegenerative disease with bradykinesia, stiffness, resting tremor, and postural instability, known as motor symptoms^1^. These individuals may also have non-motor symptoms, such as cognitive, neuropsychiatric, autonomic, and sensory disturbances^2^.

Speech disorders are experienced by about 90% of individuals with PD^3^ and result from a combination of motor deficits, especially rigidity and bradykinesia, and non-motor deficits, such as changes in auditory perception and deficit of movement programming^4^.

The theory of the four-level framework of speech sensorimotor control proposes different stages of speech production involving other neural structures. These phases are identified as the linguistic-symbolic plan (non-motor), motor planning, motor programming and execution^5^.

The linguistic (non-motor) plane is related to access to linguistic symbols.

Speech motor planning is an interface step between phonological planning for the selection and sequencing of phonemes and the preparation of impulses to be transmitted to the motor system^6^. It involves a central motor plan developed during mother tongue acquisition and neural representation areas for transforming desired movements into motor commands required to achieve the goal of these movements^7^.

Changes in speech planning include slowness, slurred speech, distortions, vocal monotony, and prosody disorders. In general, motor planning occurs at the highest level of sensorimotor speech control, while motor programming is considered a transitional phase between planning and execution^6^.

The programming phase is that which determines the spatiotemporal force dimensions, as well as the amount of muscle tension required, the specific speed, direction, and range for the movement of articulatory, phonatory, and respiratory muscles during speech. The characteristics of such motor programs are influenced by circumstantial factors (e.g., the need to speak louder or faster) and by linguistic factors that include suprasegmental features of an utterance such as intonation, tonicity, and duration^8^. The sensorimotor cortex, lateral cerebellum, and basal ganglia mediate it. More specifically, studies indicate that the selection and activation of appropriate motor programs for a particular context is one of the primary functions of the basal ganglia^9^. Disorders in the basal ganglia can cause a breakdown in the mechanisms responsible for automatic habitual performance, leading to delayed speech initiation and temporal inaccuracies^10^. Studies like this one demonstrate the involvement of these structures in the programming and execution of speech-motor control, reinforcing that hypokinetic dysarthria, commonly found in PD, cannot be understood only as an execution disorder. Spencer and Rogers demonstrated that individuals with hypokinetic dysarthria due to Parkinson's disease are unable to maintain a programmed reaction or rapidly change motor responses during speech, showing that these changes are not just impairments in the execution of movements^11^.

Finally, in the execution phase, the hierarchy of plans and programs is transformed into unlearned automatic motor tuning^6^.

Speech sensorimotor tasks performed in individuals with PKS suggest that these patients present disorders in the execution planes (such as decreased maximum phonation time and reduced articulation strength) and in speech programming (such as syllable repetitions and episodes of accelerated speech)^12^.

Voice and speech alterations include reduced voice volume, frequency fluctuations, breathiness, tremors, hoarseness, and articulatory imprecision. These changes are known as parkinsonian dysarthria or hypokinetic dysarthria^5,13^.

Speech and voice manifestations, especially hypophonia, are among the first manifestations of PD^14^.

In addition to hypokinetic dysarthria, another speech disorder observed in individuals with PD is disfluency, which different terms can represent, such as repetitive speech, stuttering, festination, or palilalia. It is characterized by involuntary repetitions, prolongations, and accelerations, resulting in difficulties controlling the rhythm and fluency of speech^15^.

The most prominent disfluency type observed in patients with PD is syllable repetition, a characteristic more related to speech programming. Thus, dysfluency or repetitive speech is unrelated to the disease's motor execution deficits; disfluency and global cognitive deterioration seem correlated^16^.

Studies also show that failures in the auditory perceptual aspects of speech can result in poor control of its production^17^.

PD individuals often show significant differences from unimpaired speakers in auditory speech monitoring. A common interpretation is that when individuals with PD are asked to produce speech with normal loudness (as judged by a speech-language pathologist), they perceive themselves as shouting or having abnormally loud speech. In addition, while listening at a given distance from a loudspeaker, individuals with PD estimated the loudness level to be significantly greater than that estimated by healthy control participants^18–20^.

In a preliminary study, it was observed that increased frequency-following neural responses related to fundamental frequency during the perception of speech in individuals with PD compared to age- and gender-matched control participants. These findings provide a neural basis for the sensory processing deficits of vocal pitch and loudness at the brainstem level in this population^21–23^.

Impaired modulation of sensory information may be one factor in the manifestations of speech deficits in individuals with PD^24^.

Auditory feedback is decoded in cortical areas, especially frontal and temporal lobes. These areas are related to higher cognitive functions such as attention and working memory, which are essential in speech-motor control^25^. A study carried out by Liu et al.^26^ showed a more significant impairment of attention and memory functions in a group of individuals with motor speech disorders when compared to a group of individuals who did not have speech disorders, suggesting a correlation between cognitive deficits and motor disorders speech in individuals with PD.

Cognitive changes may be present from the early stages of the disease, but the clinical picture tends to predominate as the disease progresses. About 40% of patients with PD have cognitive deficits in several cognitive domains, including attention, working memory and executive functions, language, visuospatial skills, and episodic memory. In later stages of the disease, cognitive decline and behavioral disturbances occur to determine clinically relevant PD-associated dementia. Part of these alterations is attributed to a dopamine-dependent dysfunction of frontostriatal pathways. Still, there is considerable heterogeneity and the influence of other neurotransmitter systems, such as the cholinergic, mainly responsible for the dementia syndrome in PD^27^.

Among the cognitive changes, executive dysfunction or dysexecutive syndrome occurs most frequently, especially in the early stages of the disease^27–38^.

Patients with PD have more difficulty forming concepts and establishing rules while performing the task. They are more inflexible and have reduced performance in competition and attentional maintenance activities^36,37^.

Higher cognitive functions have been the subject of studies evaluating the pathophysiological mechanism of abnormal speech control; however, the correlation of cognitive deficits with speech disorders still needs to be well elucidated.

A study by Wolff and Benge showed a correlation between day-to-day language difficulties, worse cognition, difficulty with daily activities, and increased motor dysfunction. ADL difficulties correlate with functional, motor, and cognitive status, even with mild functional declines predictive of everyday language difficulties^39^. Smith et al. showed that patients with PD had more pauses in utterances, fewer words per minute, and a lower percentage of well-formed sentences than controls^40^. Barbosa et al. showed a strong correlation between verbal fluency tests (semantic and phonemic) and the Trail Making Test (TMT); with patients spending more time on this task and fewer words in the phonemic/semantic verbal fluency tests and fewer syllables in the diadochokinetic test^41^.

Thus, the objective of this study was to compare the groups with and without speech disorders (including planning, programming, and execution deficits) with global cognitive assessment battery (ACE-III), neuropsychological tests, and demographic and clinical data (time of disease progression, cognitive subgroup, functionality, and severity of motor symptoms).

## METHODS

A retrospective, cross-sectional study was performed with PD patients coming from the neurological rehabilitation program of the SARAH Network of Rehabilitation Hospitals, Unit of Salvador, state of Bahia. The diagnosis of PD was defined by neurologists by means of a specialized clinical examination and according to the criteria suggested by the British Parkinson's Disease Society Brain Bank Diagnostic Criteria^42^. Data collection was between January and July 2022. A period of three months was established between cognitive and speech assessment.

The clinical group was aged over 40 years, had at least four years of schooling, had no psychiatric disorders or history of substance use and/or abuse, cerebrovascular disease, and/or other clinical conditions that could impair mental status and interfere with cognitive performance. Patients who used medications that could interfere with cognitive functioning, such as benzodiazepines and tricyclic antidepressants, were excluded.

### Cognitive assessment

Cognitive data from previous research, approved by the Research Ethics Committee of the Department of Neurology of the University of São Paulo and the SARAH Network of Rehabilitation Hospitals (Certificate of Presentation for Ethical Appreciation — CAAE: 57521316.8.0000.0022), were used. In this study, patients were evaluated using the Addenbrooke Cognitive Examination, third version (level I) (ACE-III), and neuropsychological tests (level II), according to the Movement Disorders Society guidelines^43^.

Cognitive functions were assessed by a neuropsychologist using a comprehensive battery of tests: Digit Span forward and backward (WAIS-III), Corsi Block-Tapping Test, Mental Control (WMS-R), Rey Auditory-Verbal Learning Test (RAVLT), Rey Complex Figure (RCF), TMT-A, TMT-B, TMT-Δ, Phonemic Verbal Fluency (FAS), with F, A, and S letters.

These tests were performed on patients in the “ON phase” of the medication.

After the neuropsychological assessment, patients were divided, depending on their results, into three subgroups: normal cognition in PD (NC-PD), mild cognitive impairment due to PD (MCI-PD), dementia due to PD (D-PD), as per myelodysplastic syndromes (MDS) guidelines^43,44^. Raw data were converted to Z-score. Those who scored 1.5 standard deviation below or above the mean (depending on the test) for their age and education on neuropsychological battery tests and normal activities of daily living (Functional Activities Questionnaire — FAQ <5) were diagnosed as MCI-PD. For the diagnosis of dementia, loss of functionality was considered FAQ >5 and/or Informant Questionnaire on Cognitive Decline in the Elderly — IQCODE >3.41. Subjects were classified by a professional with experience in cognitive neurology who was blinded to the patient's information.

### Speech assessment

The speech subsystems of breathing, phonation, articulation, resonance, and prosody, as well as fluency, were evaluated through the tests of sustained vowel emission, velar movement, oral and nasal emission, the rapid repetition of syllables, spontaneous speech, based on the protocol for evaluation of acquired speech disorders in Parkinson's disease (PADAF)^45^, that involves items to assess breathing, phonation, resonance, articulation, and prosody. After speech assessment, the group was stratified into two subgroups:

- Without speech disorders and
- With speech disorders.

For group stratification, we considered individuals with speech disorders to be those who had variations in the subsystems that characterize the most common alterations for the study population, cited in the literature: reduced vocal intensity, monotonous voice, intonation alteration, restricted modulation, speech disorder, breathy hoarse voice quality, articulatory imprecision, festination^4,16,45^.

### Data analysis

Data were analyzed using a statistical technique with the software Statistical Package for the Social Sciences (SPSS), version 15.0. The variables were analyzed by the chi-square test, Student's t-test, and Mann-Whitney test, according to the data type (categorical or continuous) and its distribution (through normality and homogeneity tests) when the two groups were analyzed.

Inferential tests (chi-square or Mann-Whitney) were used to compare the two subgroups regarding speech assessment, cognitive aspects, functional measures, and cognitive complaints. The groups with and without speech disorders were compared in terms of the global cognitive assessment battery (ACE-III), neuropsychological tests, and demographic and clinical data (time of disease progression, cognitive subgroup, functionality, and severity of motor symptoms).

To minimize confounding factors and/or biases in the results, secondary analyses were performed, such as by forming subgroups according to clinical variables (time of disease progression and score on the Hoehn and Yahr scale — H&Y), functional (FAQ and IQCODE) and demographic (age and level of education), to increase the study's internal validity.

Associations with p<0.05, that is, a level of significance of 5%, were considered significant.

## RESULTS

A total of 150 patients were included in this study, 104 men and 46 women, 63.58 (8.81) years of age, 11.03 (4.00) years of schooling, and 6.61 (4.69) years of disease progression, with a higher proportion of individuals (86, or 57.33%) in stage I–II of H&Y (Table 1).

**Table 1:** Demographic and clinical characteristics of the sample.

Characteristics		n=150 (%)
Sex		
	Male	104 (69)
	Female	46 (31)
Age (years)		63.58 (8.81)
Education, years		11.03 (4)
Disease duration, years		6.61 (4.69)
Hoehn & Yahr scale (%)		
	I–II	86 (57.33)
	III–IV	64 (42.67)
	BDI	5.92 (4.37)
	BAI	3.13 (3.04)
	FAQ	2.59 (3.12)
	IQCODE	3.44 (0.63)

Abbreviations: BDI, Beck's Depression Inventory; BAI, Beck's Anxiety Inventory; FAQ, Functional Activities Questionnaire; IQCODE, Informant Questionnaire on Cognitive Decline in the Elderly.

Speech disorders were identified in 118 patients, of which 109 (92%) were diagnosed with mild hypokinetic dysarthria, four with moderate hypokinetic dysarthria (3.38%), and five with festination (4.23%). In the analysis of the groups of individuals diagnosed with hypokinetic dysarthria related to speech components, changes were observed mainly in phonation, with impairment of intensity and maximum phonation time, followed by variations in articulation with articulatory inaccuracies, as well as in prosody with monotonous emissions and nasal resonance.

Sample homogeneity was maintained when analyzed in subgroups with and without speech disorders (Table 2). When the subgroups were stratified using the IQCODE and H&Y scale, a statistically significant difference was observed (p=0.033 and p=0.031 respectively), with worse performance in the subgroup with speech disorders. Regarding the other clinical aspects (disease duration, cognitive subgroup and functionality), no statistically significant difference was observed between the subgroups.

**Table 2:** Clinical and demographic characteristics of the subgroups with and without speech changes.

Variables		Without speech change (n=32)	With speech change (n=118)	p-value
Sex (%)				
	Male	21 (66)	83 (70)	0.608
	Female	11 (34)	35 (30)	
Education		12.0 (6.8)	12.0 (4.3)	0.869
Age		61.28 (9.42)	64.20 (8.57)	0.096
Disease duration (years)		5.0 (6.0)	5.0 (7.0)	0.936
Cognitive profile (%)				
	MCI-PD	24 (75)	80 (67)	0.249
	D-PD	5 (16)	17 (14)	
	NC-PD	3 (9)	21 (17)	
H&Y scale				
	I	13 (40)	12 (10)	0.031
	II	11 (34)	61 (51)	
	III	17 (55)	38 (32)	
	IV	2 (2)	7 (6)	
IQCODE				
	<3.41	26 (81)	72 (61)	0.033
	≥3.41	6 (19)	46 (40)	
FAQ				
	<5	24 (75)	93 (79)	0.644
	≥5	8 (85)	25 (21)	

Abbreviations: MCI-PD, mild cognitive impairment due to Parkinson's disease; D-PD, dementia due to Parkinson's disease; NC-PD, normal cognition in Parkinson's disease; H&Y, Hoehn & Yahr Scale; IQCODE, Informant Questionnaire on Cognitive Decline in the Elderly; FAQ, Functional Activities Questionnaire.

Significant differences were identified when comparing the subgroups with and without speech disorders with data from cognitive assessment (global and by cognitive domains) in TMT-Δ and a trend of difference in TMT-B (Table 3).

**Table 3:** Comparison between subgroups with and without speech alterations on cognitive tests and affective scales.

Variables	Without speech change (n=32)	With speech change (n=118)	p-value
	Mean (SD)	Mean (SD)	
Mental Control (WMS)	6.0 (1.0)	6.0 (2.0)	0.300
RCF (copy)	32.0 (13.5)	30.5 (13.0)	0.656
RCF Time (copy)	300.5 (151.5)	332.5 (307.0)	0.071
RCF (long-delay recall)	12.8 (10.5)	12.3 (10.1)	0.822
RAVLT (total-A)	36.5 (16.8)	34.0 (16.3)	0.342
RAVLT (recall B)	4.0 (2.8)	7.0 (4.0)	0.342
RAVLT (post interference)	7.0 (4.0)	6.0 (4.0)	0.143
RAVLT (delayed recall 30')	6.0 (5.0)	5.0 (5.0)	0.745
RAVLT (recognition)	13.0 (2.0)	12.5 (3.0)	0.619
Verbal Fluency (FAS)	28.5 (16.5)	25.0 (17.3)	0.267
Digit Span (forward)	6.0 (2.0)	5.0 (2.0)	0.448
Digit Span (backward)	4.0 (1.0)	3.0 (1.0)	0.167
Corsi Block (forward)	4.5 (1.0)	5.0 (2.0)	0.288
Corsi Block (backward)	4.0 (0.0)	4.0 (2.0)	0.349
TMT-A (sec)	74.5 (51.3)	131.1 (55.5)	0.064
TMT-B (sec)	191.5 (158.5)	399.0 (153.5)	0.059
TMT-Δ (B-A)	111.50 (69.46)	266.58 (90.32)	0.048
ACE-III (total)	84.0 (19.0)	83.5 (13.3)	0.838
Attention/Orientation	17.0 (4.0)	15.0 (3.0)	0.364
Memory	19.5 (8.0)	18.5 (7.5)	0.892
Fluency	10.0 (3.0)	8.0 (3.0)	0.381
Language	26.0 (2.0)	24.0 (2.0)	0.810
Visuospatial	15.0 (4.0)	16.5 (6.0)	0.576
BDI	4.5 (5.8)	5.0 (6.3)	0.223
BAI	2.0 (3.8)	6.0 (3.0)	0.561

Abbreviations: RCF, Rey Complex Figure; RAVLT, Rey Auditory Verbal Learning Test; TMT, Trail Making Test; ACE-III, Addenbrooke's Cognitive Examination III; BDI, Beck's Depression Inventory; BAI, Beck's Anxiety Inventory.

## DISCUSSION

This study evaluated the comparison between speech disorders and cognitive alterations in individuals with PD, specifically checking the analysis among the cognitive subgroups.

Analyzing the sociodemographic data, a homogeneity between the subgroups with and without speech disorders was observed, even with the stratification of the sample in terms of sex, mean age, and schooling, strengthening our results, together with extensive and reliable batteries for cognitive disorders in this population.

The literature cites the auditory-perceptual assessment as the gold standard for the description, quantification, and differential diagnosis of dysarthria^46^.

Several studies indicate dysarthria is the most frequent speech disorder in individuals with PD^47^. Hypokinetic or parkinsonian dysarthria may be caused by motor planning deficiency and is associated not only with muscle stiffness, bradykinesia, and tremors but with other motor and gait alterations^48,49^. Speech can be affected due to disorders in the basal ganglia, as these structures are involved in planning, programming, and performing motor tasks. It was hypothesized that they play an essential role in articulatory control, including the selection of motor programs, sensorial execution, and feedback^50–52^.

In the present study, individuals with speech disorders showed phonation, breathing, articulation, resonance, and prosody variations that characterized mild to moderate hypokinetic dysarthria or festination. This study aimed not to further characterize these speech alterations, but to compare speech disorders (including planning, programming, and execution deficits) and cognitive alterations in individuals with PD.

In the present study, individuals with speech disorders presented a greater severity of motor symptoms with a statistically significant difference in the H&Y scale, just as these patients had more complaints of cognitive alteration (assessed by the IQCODE questionnaire). The sample, however, consisted of patients diagnosed with MCI-PD, without functional impairment and/or more significant impairment of motor symptoms.

In the analysis of the subgroup with a speech disorder, there was also more significant impairment in tests assessing executive functions, indicated by the worst performance in the TMT-B and mainly TMT-Δ. Parts B and Δ require mental flexibility, task switching, response inhibition, and working memory, which assesses cognitive-motor dual-task performance^53^. The TMT-B is a more sensitive test for assessing cognitive functioning, especially measures of divided and alternating attention, suggesting worse performance in this cognitive domain in patients with speech disorders when compared to those without speech disorders. This result was not observed in the other tests since they are related to other aspects of cognitive functioning. It should be noted that most patients had a diagnosis of MCI-PD and stages I–II of H&Y, with dysfunction in attentional and executive measures being more prevalent.

These findings can also be justified since executive function depends on frontal structures, which are impaired in people with PD due to dopamine depletion in nigrostriatal projections, especially in the early stages of the disease, as throughout the course of the illness other neurotransmitter systems are altered^54^.

Individuals with PD may fail to perform tasks with high demands on executive functions when problem-solving requires the subject to plan and execute a strategy using only the source of internal cues to guide behavior, signaling a reduction in pathways responsible for executive functioning. Frontostriatal dysfunction is thought to occur in PD due to a lack of dopaminergic activity in the striatum, leading to impairment of cognitive and associative striatal circuits^40^. These changes can be observed from the early stages of the disease or even in patients not yet treated.^42^ In this study, most of the sample had an average of five years of symptoms.

The limitations of this study were:

- The highest proportion of individuals were in stages I–II of H&Y — it is therefore essential to emphasize that these analyses cannot be generalized to the other stages of the disease progression;
- It is a retrospective study;
- There is an absence of a control group;
- There is discrepancy in the distribution of individuals between subgroups with and without speech disorder; and
- Speech manifestations are not characterized.

In conclusion, individuals with speech disorders showed more severe motor symptoms and had more frequent cognitive complaints. This study showed a statistically significant difference in tests assessing executive attention aspects. The subgroup with speech disorders had more substantial impairment in tests assessing executive functions, worse severity of motor symptoms (H&Y scale) and cognitive complaints (IQCODE).

This study provides data that allow a better understanding of the interactions between speech disorders and cognitive functions, favoring therapeutic strategies in population under study.
